# Participant Engagement and Reactance to a Short, Animated Video About Added Sugars: Web-based Randomized Controlled Trial

**DOI:** 10.2196/29669

**Published:** 2022-01-24

**Authors:** Caterina Favaretti, Alain Vandormael, Violetta Hachaturyan, Merlin Greuel, Jennifer Gates, Till Bärnighausen, Maya Adam

**Affiliations:** 1 Heidelberg Institute of Global Health Heidelberg University Heidelberg Germany; 2 Icahn School of Medicine Mount Sinai New York, NY United States; 3 Africa Health Research Institute Wellcome Trust KwaZulu-Natal South Africa; 4 Harvard Center for Population and Development Studies Harvard University Cambridge, MA United States; 5 Department of Pediatrics School of Medicine Stanford University Stanford, CA United States

**Keywords:** added sugar, prevention, sugar consumption, short and animated story-based video, informational video, randomized controlled trial, social media, participant engagement, health promotion, United Kingdom, entertainment, patient education, healthy eating, preventive health, health messaging

## Abstract

**Background:**

Short, animated story-based (SAS) videos are a novel and promising strategy for promoting health behaviors. To gain traction as an effective health communication tool, SAS videos must demonstrate their potential to engage a diverse and global audience. In this study, we evaluate engagement with a SAS video about the consumption of added sugars, which is narrated by a child (a nonthreatening character), a mother (a neutral layperson), or a physician (a medical expert).

**Objective:**

This study aims to (1) assess whether engagement with the sugar intervention video differs by narrator type (child, mother, physician) and trait proneness to reactance and (2) assess whether the demographic characteristics of the participants (age, gender, education status) are associated with different engagement profiles with the sugar intervention video.

**Methods:**

In December 2020, after 4013 participants from the United Kingdom completed our randomized controlled trial, we offered participants assigned to the placebo arms (n=1591, 39.65%) the choice to watch the sugar intervention video (without additional compensation) as posttrial access to treatment. We measured engagement as the time that participants chose to watch the 3.42-minute video and collected data on age, gender, education status, and trait reactance proneness. Using ordinary least squares regression, we quantified the association of the demographic characteristics and trait reactance proneness with the sugar video view time.

**Results:**

Overall, 66.43% (n=1047) of the 1576 participants in the 2 placebo arms voluntarily watched the sugar intervention video. The mean view time was 116.35 (52.4%) of 222 seconds. Results show that view times did not differ by narrator (child, mother, physician) and that older participants (aged 25-59 years, mean = 125.2 seconds) watched the sugar video longer than younger adults (aged 18-25 years, mean = 83.4 seconds). View time remained consistent across education levels. Participants with low trait reactance (mean = 119.3 seconds) watched the intervention video longer than high-trait-reactance participants (mean = 95.3 seconds), although this association did not differ by narrator type.

**Conclusions:**

The majority of participants in our study voluntarily watched more than half of the sugar intervention video, which is a promising finding. Our results suggest that SAS videos may need to be shorter than 2 minutes to engage people who are young or have high trait proneness to reactance. We also found that the choice of narrator (child, mother, or physician) for our video did not significantly affect participant engagement. Future videos, aimed at reaching diverse audiences, could be customized for different age groups, where appropriate.

**Trial Registration:**

German Clinical Trials Register DRKS00022340; https://www.drks.de/drks_web/navigate.do?navigationId=trial.HTML&TRIAL_ID=DRKS00022340

**International Registered Report Identifier (IRRID):**

RR2-10.2196/25343

## Introduction

Engaging the public in the care and maintenance of their own health constitutes a longstanding challenge for health communicators, health educators, and public health agencies worldwide [1,2]. Innovative strategies, including the use of pictures [3], digital storytelling, and entertainment-education [4], have all shown promise for increasing engagement in public health campaigns. Research has shown that packaging health recommendations in a relatable story can be more effective than traditional media approaches that frame health messages as informational arguments [5]. More recently, social media has emerged as an important platform for communicating evidence-based health messages and potentially improving health outcomes [2,6]. Aligned with this innovative direction, short, animated story-based (SAS) videos draw from entertainment-education media, communication theory, and the animated entertainment industry to promote compelling, evidence-based health messages that are optimized for “viral spread” over social media channels [7,8]. Under 4 minutes in length and using culturally de-identified character portrayals, SAS videos are designed to be accessible and adaptable across different global regions, languages, and literacy levels [7,9]. However, to gain further traction as a health communication tool, SAS videos must demonstrate their potential to catalyze engagement across diverse audiences.

**Figure 1:**
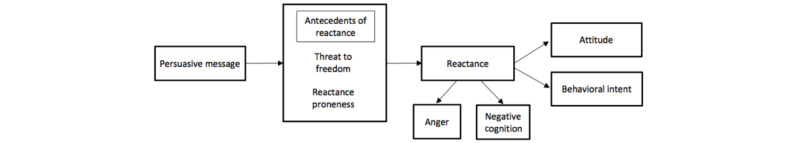
The Intertwined Process Cognitive-Affective Model of reactance [11],[12].

As with all persuasion strategies, optimal engagement with SAS videos may be limited by a motivation to reject the health message—a phenomenon known as reactance [10]. As a theoretical construct, reactance consists of 4 main components: (1) freedom, which individuals possess insofar as they are aware of it and can enact it; (2) threat to freedom, which is anything that makes it difficult to enact that freedom; (3) reactance, which is the motivation to reestablish the freedom if that freedom is eliminated or threatened with elimination; and (4) direct restoration, which involves the freedom of the individual to perform a forbidden act [11]. In the communications literature, Dillard and Shen [11] and Zhang [12] have proposed the Intertwined Process Cognitive-Affective Model, which describes the pathways through which a persuasive message can provoke reactance (Figure 1). The model includes 2 antecedents to reactance: threat to freedom and trait reactance proneness, which is a personal trait or propensity to experience reactance [13], reactance itself (comprising anger and negative cognition), and its outcomes (attitude and behavioral intent). Previous research on reactance in the health sciences has led to the development of several strategies to reduce reactance in areas such as the use of e-cigarettes [14], littering [15], alcohol [16], and eating behaviors [17], among others [5,18-22]. Of these strategies, we are most interested in the narrator’s characteristics (eg, the claim to expertise, intended motive, the threat level of the narrator) that are likely to arouse reactance to health messages.

In a recent study, we investigated whether a child narrator reduced reactance to a SAS video about the consumption of added sugars [23]. In the video, the 2 main characters, a mother and her preadolescent daughter, engage in food-related activities, such as shopping for groceries and cooking dinner. Through a narrative, they present educational content on the health problems associated with the addition of excess sugars in commonly available foods. Using a web-based experiment platform, we randomized 4013 participants to the same sugar video narrated by the daughter (a nonthreatening character), the daughter’s mother (a neutral layperson), or the family physician (an expert with medical authority). We then compared the differences in reactance to the 3 narrators relative to a SAS video with a health message about sunscreen use (the content placebo) and a SAS video with a non-health-related message about earthquakes (the placebo). We hypothesized that the child narrator would arouse the least reactance to the sugar intervention message.

In this study, we investigate the role of trait reactance proneness and demographic factors in voluntary engagement with the sugar intervention video narrated by the child, the mother, or the physician. The participants (n=1576) are those who were initially randomized to the content placebo (the sunscreen SAS video) or the placebo (the earthquake SAS video) arms in the main trial and who were then offered the intervention video as posttrial access to treatment [24]. We define engagement as the duration of time that the participants spent watching the 3.42-minute intervention video. We hypothesized that participants with lower trait reactance proneness would spend more time watching the intervention videos, with the child-narrated video having the longest view time (assuming it would arouse the least reactance). In addition, given that SAS videos are designed for social media, we hypothesized that younger participants (aged 18-24 years) would watch the intervention video longer than older participants (aged 25-59 years). Findings from our study could inform the future design and delivery of effective, spreadable SAS videos aimed at promoting health in diverse audiences.

## Methods

### Study Design and Participants

**Figure 2:**
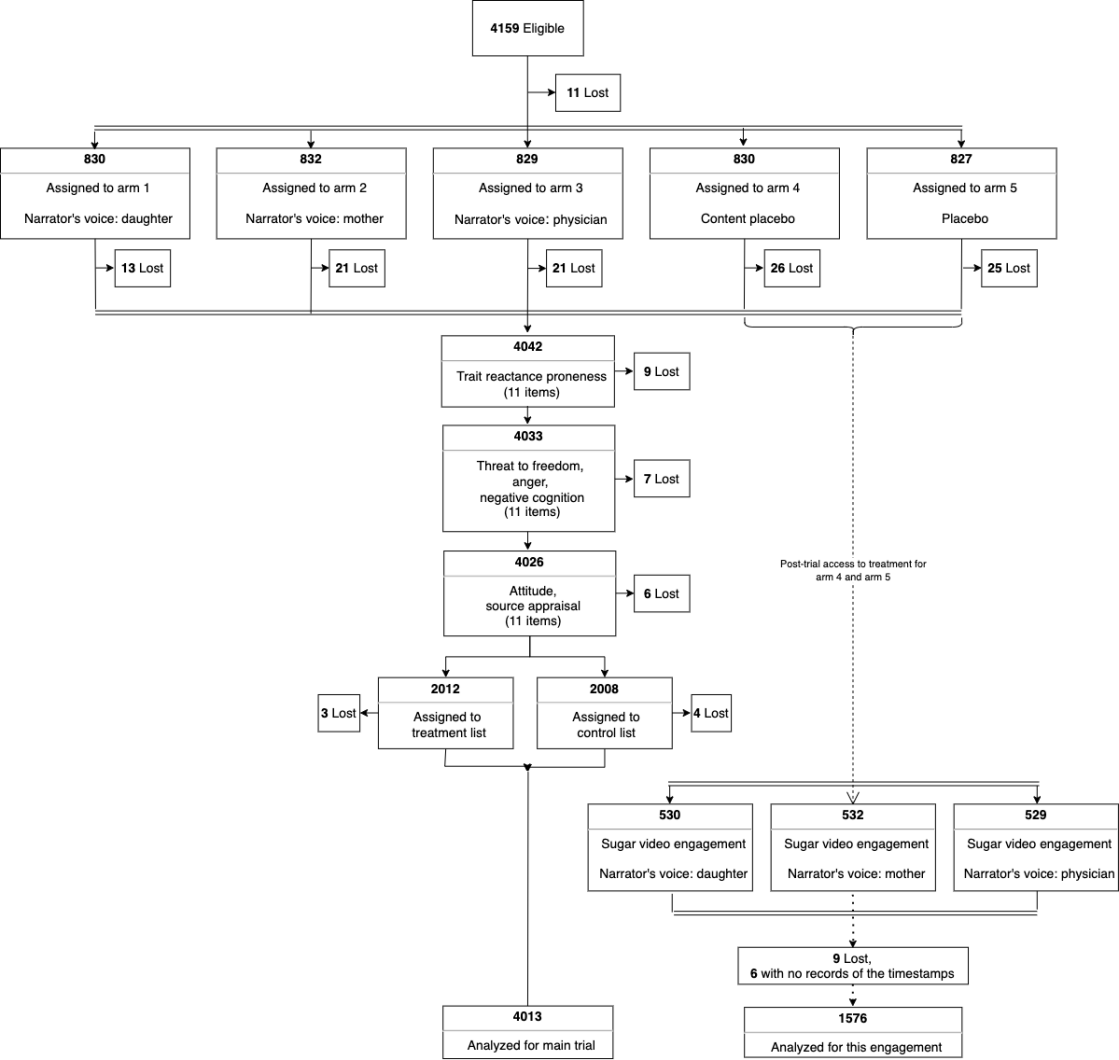
Flowchart depicting the study methodology.

This was a randomized controlled trial (RCT) with posttrial access to the treatment stage [23]. In the main trial, participants were randomly assigned (1:1:1:1:1) to 3 different intervention arms (arms 1-3), a content placebo arm (arm 4), and a placebo arm (arm 5). In each intervention arm, respondents watched a SAS video about sugar consumption, narrated by 3 different voices: a preadolescent daughter (arm 1), the daughter’s mother (arm 2), and the family physician (arm 3). In the content placebo arm, participants watched a SAS video with a non-sugar-related message about sunscreen use; in the placebo arm, participants watched a non-health-related video about earthquakes (Figure 2). At the end of the trial, participants randomized to the content placebo and placebo arms were given the option to watch the sugar intervention video. If these participants agreed, they were then randomized 1:1:1 to the sugar video narrated by the child, the mother, or the physician.

Both trials (main and post) were hosted and run on the Gorilla platform (Cauldron Science Limited) [25] and participants were recruited through Prolific (Prolific Academic Ltd) [26]. Inclusion criteria included being between the ages of 18 and 59 years (male, female, or other), being able to speak English, and residing in the United Kingdom. More details on the sample size determination can be found in Multimedia Appendix 1. The study and its outcomes were registered with the German Clinical Trials Register [27] on July 24, 2020 (#DRKS00022340). Ethical approval was obtained from the Heidelberg University’s ethics committee on March 18, 2020 (#S-088/2020). No harm or adverse events were observed, given the online format of the trial.

### Randomization and Blinding

The Gorilla algorithm randomly assigned participants to the 5 arms in the main trial and to the 3 intervention arms in the posttrial stage. Since the recruitment took place on the Prolific platform, it was not possible to identify or link data back to the participants. Participants responded to the survey questions and submitted their responses anonymously through the Gorilla platform. Both the study subjects and the investigators had no knowledge regarding the allocation status of the participants.

### Informed Consent

All participants underwent a process of informed consent on the Prolific platform. The consent form explained the purpose of the study, the risks and benefits of the research, and how to contact the study investigators. By clicking the link, participants agreed to participate in our study and were redirected to the Gorilla platform, where additional information was given. Participants could leave the research study at any time.

### Procedures

Here, we provide some basic details of the main trial to give context to our posttrial study. Further details of the main trial and its procedures can be found elsewhere [23].

At the beginning of the main trial, participants were asked to answer demographic questions about their age, gender, and highest educational attainment. Participants were then randomized to the sugar intervention arm, the content placebo arm, or the placebo arm, where they watched a SAS video from start to finish.

The sugar intervention video was narrated in English, with a duration of 3 minutes and 42 seconds. Its aim was to boost knowledge about the health consequences of consuming added sugars [28-30]. The video presented the WHO recommendations for daily sugar consumption, the health risks associated with excess consumption, and some strategies for reducing sugar in an individual's daily diet. The characters were deliberately represented without distinguishable cultural identifiers, to enhance cross-cultural appeal, while the soundtrack was designed to arouse emotion and enhance engagement. We, the coauthors, decided to compare the child narrator with the mother and family physician narrators. In the content placebo arm, respondents watched an animated video delivering a non-sugar-related health message about tanning and the use of sunscreen [31]. In the placebo arm, participants watched a non-health-related video about the causes and characteristics of earthquakes [32]. Both content placebo and placebo videos were animated, short (3.42 minutes), and narrated by a single character. We chose these nonintervention videos to be as similar as possible to the sugar intervention video but with no sugar content (the content placebo video about sunscreen use) and no health message (the placebo about earthquakes). Although both placebo and content placebo videos were chosen with caution, they were taken from external sources, and we, therefore, could not modify the design of those videos. After watching the SAS video, participants answered questions about their proneness to trait reactance.

For this study, participants who were randomized to the content placebo video or placebo video were then given the option to watch the sugar intervention video (posttrial access to treatment). Participants could watch the sugar video or end the study without watching the sugar video. If participants chose to watch the sugar video, they were asked on the next page to click the Play button or click the Finish button at any time to end the survey. The participants were informed that they would not be compensated for the additional time taken to watch the sugar video.

### Measures

The primary outcome of this study was participant engagement, measured as the total time (in seconds) spent watching the SAS sugar video. We also collected data on the participants’ age, gender, and educational status. We further considered the role of the participants’ propensity toward reactance and its effect on view time. To measure trait reactance proneness, participants answered 11 questions based on the Hong Psychological Reactance Scale [27]. The questions comprised 4 major factors: emotional response to restricted choice, reactance to compliance, resisting influence from others, and reactance to advice and recommendations. Possible responses were arranged along a 5-point scale, anchored by strongly disagree (1) and strongly agree (5).

### Statistical Analysis

To quantify the participants’ engagement, we used the graphical experiment builder in Gorilla that records a timestamp whenever a new screen is displayed. In our case, Gorilla registered the moment when the participant reached the instruction screen of the final task as the first timestamp, the moment when they entered the video screen as the second timestamp, and the moment when they ended the experiment as the third timestamp. Gorilla also recorded loading delays of more than 10 seconds.

Participants who spent less than 3 seconds on the video screen were grouped together with participants who did not watch the SAS video. Among participants who chose to watch the SAS video, we quantified the length of time spent watching the sugar video. We defined the dependent variable *engagement time* as the difference between the third timestamp and the second timestamp. The resulting variable was reported in seconds between 0 (ie, the respondent watched 0 seconds of the SAS video) and 222 (ie, the respondent watched the entire SAS video). We used 5 ordinary least squares regression models to investigate which sociodemographic factors and narrator’s voice were associated with engagement time. Model 1 included narrator, a categorical variable that equaled 1 if the participant was randomly assigned to watch the sugar video narrated by the preadolescent daughter, 2 if the narrator’s voice of the video was the mother’s, and 3 if it was the physician’s. Models 2-4, respectively, added age, gender, and education completed, which were all categorical variables. We included each categorical variable nonparametrically in our model as a set of dummies. Model 5 added the participants’ trait reactance proneness mean score, which is a continuous variable between 0 and 5. The methodology for calculating the participant’s trait reactance proneness mean score is described in the study protocol [23].

We dropped observations that had missing values and performed all statistical analyses using Stata software version 14.2.

## Results

### Principal Findings

Between December 9, 2020, and December 11, 2020, we recruited 4159 participants for the main RCT. The main trial design is shown in Figure 2 and described elsewhere [23]. Of the 4159 participants, 1591 (38.25%) were assigned to 1 of the 2 placebo videos, of which 15 (0.94%) had missing data. Of the final sample of 1576 participants, 957 (60.7%) were female and 504 (32%) were between the ages of 25 and 34 years. In addition, over 1292 (82%) of participants had obtained at least a bachelor’s degree. Table 1 shows the summary statistics of the sociodemographic variables by trial arm and narrator’s voice (child, mother, physician). The *P* values stem from chi-squared tests and provide evidence that the randomization was successful.

**Table 1:** Characteristics of 1576 participants from the United Kingdom, with data on engagement with a short, animated video about added sugars in a web-based RCT^a^, December 2020.

Demographics			Content placebo arm (790 observations)				Placebo arm (786 observations)		
		Narrator 1 (child), n (%)		Narrator 2 (mother), n (%)	Narrator 3 (physician), n (%)	Narrator 1 (child), n (%)		Narrator 2 (mother), n (%)	Narrator 3 (physician), n (%)
**Age (years), *P*=.68^b^**									
	18-24	57 (22.8)		64 (22.5)	63(24.7)	69(25.1)		68(28.2)	71 (26.3)
	25-34	83 (33.2)		93 (32.6)	80 (31.4)	89 (32.4)		74 (30.7)	85 (31.5)
	35-44	57 (22.8)		67 (23.5)	51 (20.0)	58 (21.1)		50 (20.7)	59 (21.8)
	45-54	37 (14.8)		41 (14.4)	49 (19.2)	41 (14.9)		36 (14.9)	39 (14.4)
	55-59	16 (6.4)		20 (7.0)	12 (4.7)	18 (6.5)		13 (5.4)	16 (5.9)
	*P* value^c^	N/A^d^		.77	N/A	N/A		.99	N/A
**Gender, *P*=.72^b^**									
	Female	155 (62.0)		167 (58.6)	153 (60.0)	179 (65.1)		142 (58.9)	161 (59.6)
	Male	95 (38.0)		117 (41.0)	98 (38.4)	92 (33.4)		98 (40.7)	107 (39.6)
	Other	0 (0.0)		1 (0.3)	4 (1.6)	4 (1.4)		1 (0.4)	2 (0.7)
	*P* value^c^	N/A		.19	N/A	N/A		.31	N/A
**Education status, *P*=.87^b^**									
	Primary school	6 (2.4)		4 (1.4)	3 (1.2)	2 (0.7)		3 (1.2)	5 (1.8)
	High school	37 (14.8)		45 (15.8)	38 (14.9)	39 (14.2)		42 (17.4)	45 (16.7)
	BA, some college	155 (62.0)		176 (61.7)	166 (65.1)	184 (66.9)		152 (63.1)	160 (59.3)
	MA/PhD	52 (20.8)		60 (21.0)	48 (18.8)	50 (18.2)		44 (18.3)	60 (22.2)
	*P* value^c^	N/A		.19	N/A	N/A		.54	N/A

^a^RCT: randomized controlled trial.

^b^The *P* value comes from a Chi-squared test comparing the distribution of the respective covariates between the two study arms.

^c^The *P* value comes from a Chi-squared test comparing the distribution of the respective covariates between the three different narrators.

^d^N/A: not applicable.

**Figure 3:**
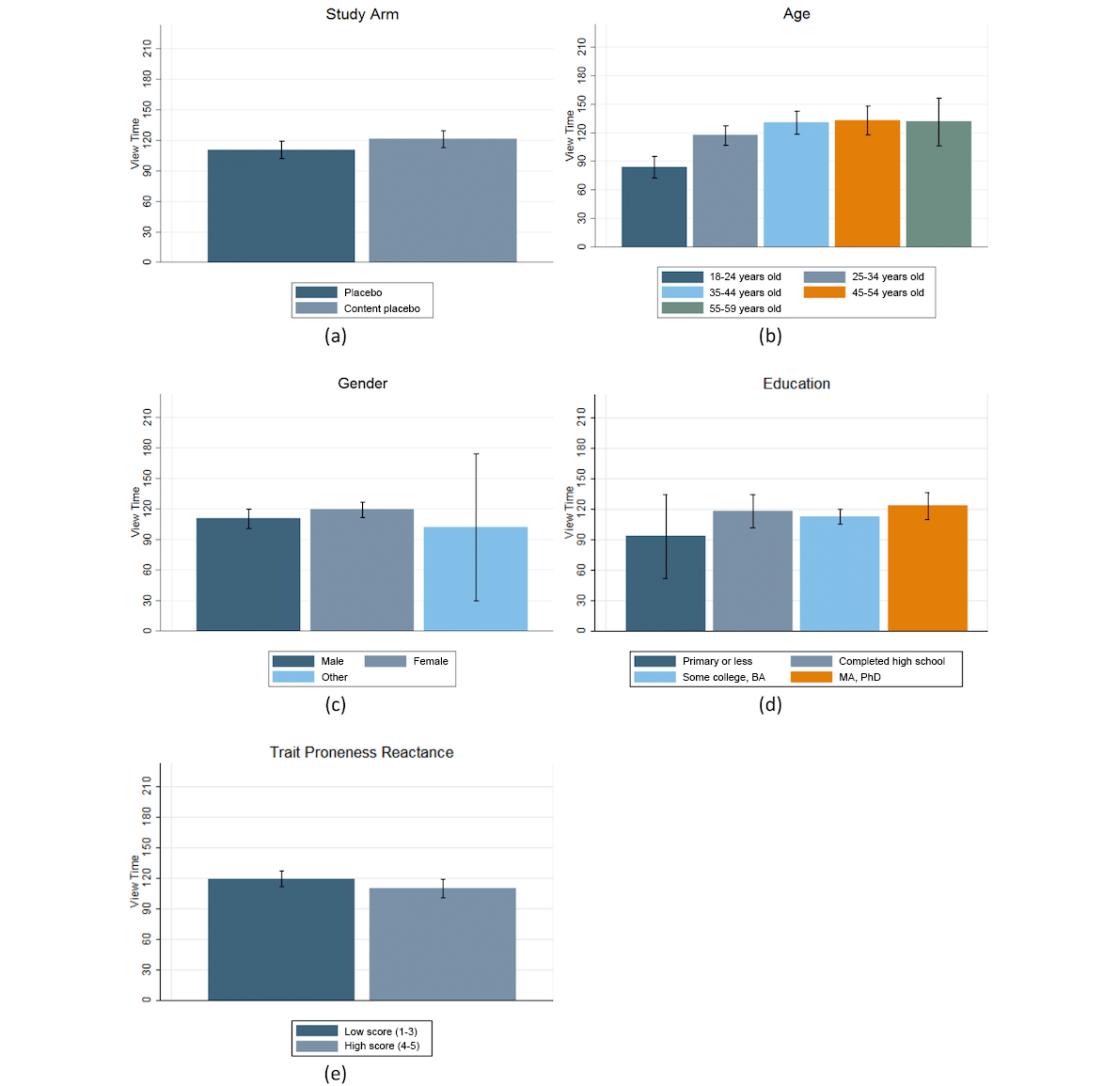
Participant view times (n=1047) of a short, animated video about sugar intake by narrator’s voice, sociodemographic characteristics, and trait proneness reactance. Note: The whiskers represent the 95% CIs of view time.

A total of 1047 (66.43%) of the 1576 participants chose to watch the sugar video. Among these participants, the average time spent watching the sugar video was 116.35 (52.4%) of 222 seconds. Figure 3 displays the average view time by the narrator’s voice (child, mother, physician), age, gender, and education status. Results show that the average view times did not significantly differ between the child, mother, and physician trial arms. Moreover, older participants (aged 25-59 years, mean = 125.2 seconds) watched the sugar video longer than younger adults (aged 18-25 years, mean = 83.4 seconds). Specifically, after adjusting for sociodemographic factors, participants aged 25-34 years watched 32.92 seconds longer than younger participants (reference category), participants aged 35-44 years watched 46.96 seconds more, participants aged 45-54 years watched 47.50 seconds more, and participants aged 55-59 years watched 46.78 seconds more (*P*<.001, Table 2, model 5). Although not statistically significant, female participants tended to watch the video almost 8 seconds longer than males (Table 2, column 5). After adjusting for our set of covariates, we observed that the view time did not significantly vary across different educational levels (Table 2, column 5). Results show that participants with higher levels of reactance proneness were likely to watch the SAS video for a shorter period. A 1 unit increase in the reactance proneness mean score was associated with a 10.86-second decrease in the SAS view time (*P*=.07, Table 2, model 5).

**Figure 4:**
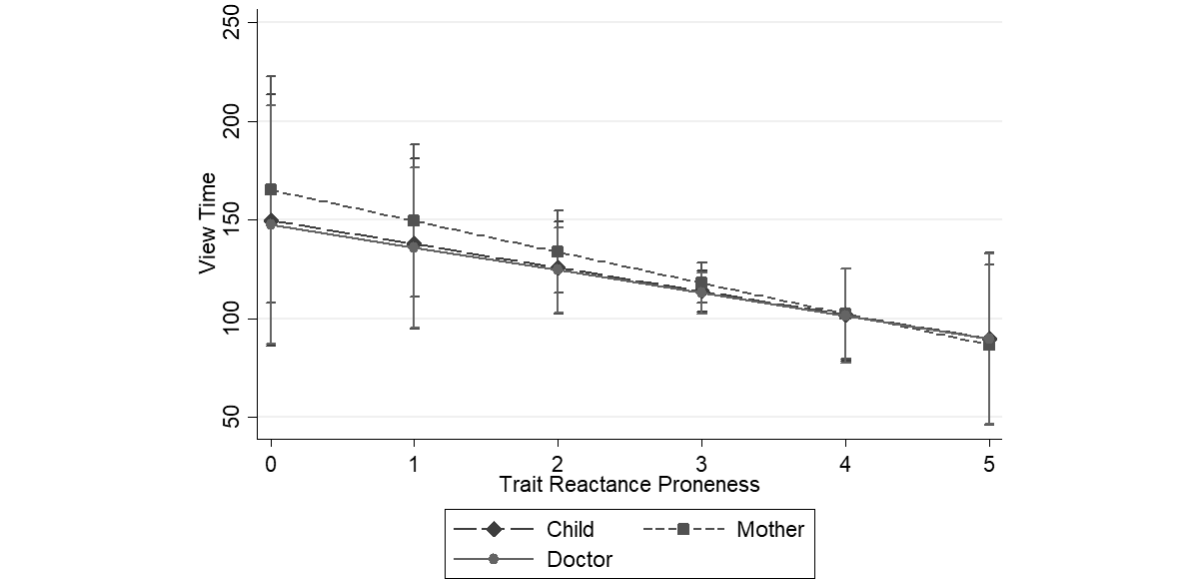
Predicted view times (n=1047) by narrator’s voice of a short, animated video about sugar intake. Note: The whiskers represent the 95% CIs at predicted trait proneness values.

To see view time as a function of trait reactance proneness for the child, mother, and physician narrators, adjusted for education level, please see Multimedia Appendix 1. The lack of significance suggests that the relationship of the reactance proneness mean score on the view time did not vary by the narrator’s voice. Figure 4 shows the interaction between trait reactance proneness and the narrator’s voice, and it reveals that participants with high trait reactance (scores of 4 or more) watched, on average, 95.3 seconds of the video, while those with low or moderate levels of trait reactance (scores of 3 or less) watched, on average, 119.3 seconds.

**Table 2:** Linear regression coefficients of factors (narrator’s voice, sociodemographic characteristics, reactance proneness) associated with engagement with a short, animated video about added sugars (n=1047).

Factors		Model 1 (SE, *P* value)	Model 2 (SE, *P* value)	Model 3 (SE, *P* value)	Model 4 (SE, *P* value)	Model 5 (SE, *P* value)
**Narrator (Ref^a^: daughter)**						
	Narrator 2: mother	5.217 (7.362, .48)	4.196 (7.250, .56)	4.820 (7.255, .51)	4.807 (7.265, .51)	3.995 (7.262, .58)
	Narrator 3: physician	0.023 (7.429, .99)	–1.080 (7.306, .88)	–0.616 (7.307, .99)	–0.849 (7.304, .91)	–1.582 (7.295, .83)
**Age (years; Ref: 18-24)**						
	25-34	—^b^	33.44 (7.873, <.001)	33.60 (7.857, <.001)	33.42 (8.022, <.001)	32.92 (8.026, <.001)
	35-44	—	46.62 (8.546, <.001)	47.41 (8.546, <.001)	47.22 (8.622, <.001)	46.96 (8.624, <.001)
	45-54	—	49.28 (9.609, <.001)	48.99 (9.591, <.001)	48.77 (9.575, <.001)	47.50 (9.592, <.001)
	55-59	—	47.80 (13.959, .001)	48.68 (13.886, <.001)	47.77 (13.977, .001)	46.78 (13.897, .001)
**Gender (Ref: female)**						
	Female	—	—	10.21 (6.109, .095	10.47 (6.134, .09)	10.02 (6.129, .102)
	Other	—	—	–6.822 (33.142, .84)	–6.874 (33.296, .84)	–7.537 (34.051, .83)
**Education status (Ref: primary school)**						
	High school	—	—	—	28.63 (22.411, .202)	25.43 (22.257, .25)
	BA, some college	—	—	—	21.54 (21.230, .31)	19.61 (21.037, .35)
	MA/PhD	—	—	—	27.02 (21.977, .22)	23.90 (21.844, .27)
**Trait reactance proneness**		—	—	—	—	–10.86 (5.871, .07)
	n	1047	1047	1047	1047	1047

^a^Ref: reference group.

^b^Not applicable.

## Discussion

### Principal Findings

In this web-based RCT, we assessed participant engagement with a SAS video about added sugar consumption. We hypothesized that participants with higher levels of trait reactance proneness would watch the SAS video for a shorter period and that younger participants (aged 18-24 years) would have higher engagement with the sugar intervention when compared with older participants (aged 25-59 years). Overall, 66.43% (1047/1576) of the participants voluntarily watched the sugar intervention video with an average view time of 116.35 (52.4%) of 222 seconds. We observed that participants with low levels of trait reactance proneness watched the video longer, whereas contrary to our expectations, older participants watched the intervention video longer than younger participants.

As stated, our results show that the majority of the 1576 participants chose to engage with the intervention video and watched, on average, more than half of the video. In recent years, an ever-increasing number of offerings, including high-budget entertainment productions, have competed to occupy our leisure time. The degree of voluntary engagement seen in this study, despite a comparatively low-budget, SAS health video, underscores the potential for this health communication modality. The engagement documented in our study also far exceeds patient engagement with print-based health communication materials distributed in health care settings [33]. In 1 such study, Williams et al [33] found that only 15% of participants reported voluntarily reading written materials provided by their doctors.

We examined the role of trait reactance proneness on participants’ view time, which has been shown to be an obstacle to successful health promotion campaigns [34]. Because individuals with high levels of reactance have a need to maintain or restore their perceived or actual personal freedoms, we assumed that high levels of reactance would negatively affect the view time. Indeed, we observed that view time was less for participants who scored high in trait reactance proneness regardless of the narrator. Specifically, the findings reveal that participants high in reactance watched, on average, less than 100 seconds of the SAS video, irrespective of the narrator. This result is in line with the literature on trait reactance proneness, which details that the outcomes of reactance are detrimental to health communication campaigns and noncompliance in instructive interventions. Bensley and Wu [35], for instance, found that high-threat messages recommending either abstinence or controlled drinking create a reactance effect, as demonstrated by negative ratings and higher consumption. It follows that individual differences in trait reactance must be clearly considered while designing an SAS intervention. Future videos should aim at being as concise as possible and potentially less than 2 minutes long in order to engage those with high levels of reactance or proneness to reactance.

In the main trial, we investigated the role of the narrator on reactance to the sugar video. Since previous studies have shown that individuals may perceive doctors as coercive or overly directive [36], we first hypothesized that a child narrator would be perceived as a nonthreatening health messenger, thereby arousing less reactance. In the main trial, we found no evidence that the child narrator attenuated reactance to the sugar reduction message when compared with the physician and mother narrators [37]. Consistent with previous results [37], our findings from the posttrial stage show that the implementation of different narrator voices did not influence participants’ view time, suggesting that their level of reactance was not altered. These findings suggest that using a child narrator may neither reduce reactance nor increase engagement with SAS videos. Other variables, such as content length, may be more important to optimize for different target audiences.

Since the SAS video was designed for rapid distribution on social media channels, we expected higher participation from younger participants (aged 18-25 years). Surprisingly, we found that older people (ages 25 years and more) watched the video, on average, longer than younger participants. This result might be explained by the perceived vulnerability among older adults, that they are more likely to suffer from health problems that are associated with an excessive consumption of added sugar. Furthermore, longer viewing times in older adults might also be connected to differences in information processing or the perceived seriousness and involvement in the study. Younger participants (ie, emerging adults in this case) are less risk averse and more accustomed to engaging with extremely short forms of content [38,39]. Another reason might be that younger people, who constantly engage with social media, might find animated health videos less entertaining or novel than older people, who spend considerably less time on social media [40]. This is echoed also in the notion that younger people have shorter attention spans, potentially driven by an increased availability of a plethora of online content, rendering them a challenging target audience [41]. This finding is consistent with the results of a recent online study we conducted on participant engagement with a short, animated video about COVID-19 prevention [42], where, too, younger participants viewed the video for a shorter amount of time, on average. This suggests that older participants, rather than younger participants, could benefit the most from SAS health videos delivering a story that unfolds a little more slowly than many contemporary social media posts. To optimally engage different target audiences, future SAS videos could be customized for different age groups.

### Strengths

A key strength of this study was the use of an RCT design, which allowed us to reduce any systematic differences and bias through randomization. In addition, the use of an online recruitment platform helped us reach a large sample size, ensuring the quality and reliability of the results. We are not aware of any other study that had such a large sample size and used a similar experimental approach to examining participant engagement in the field of public health. Arguably, this posttrial stage of our RCT enabled us to capture participants’ voluntary willingness to watch a SAS video without any financial compensation. Although this condition is similar to the real world, we acknowledge that participants’ responses may have been affected by their awareness of being in a scientific study and that their actions were being recorded for scientific purposes. Nevertheless, outside of a scientific study setting, we report anecdotal evidence of willingness to engage in our sugar intervention video. After our RCT, the child-narrated version of the video was posted on the creator’s (author MA) YouTube channel, where it reached 3700 views in the first 48 hours after its release.

### Limitations

Our study had several limitations. Given the online setting of our study, we were not able to determine whether participants actively watched the intervention video (it may have been playing in the background while the participant was engaged in other activities). Given the posttrial phase of our study, we were only able to evaluate the role of demographic factors and trait proneness reactance on participant engagement with the sugar intervention video. We acknowledge that other factors could have affected the time that participants spent watching the sugar intervention video, such as the perceived threat of the message, the perceived threat to health, the perceived risk of adopting an alternative behavior, and anger and negative cognition toward the sugar message (see Figure 1). In future research, we could address this limitation by considering how engagement with SAS videos is affected by these factors, which are typically included in health communication models and research [43-46]. Another limitation is that our online sample was relatively well educated, with 1308 of 1576 (83%) participants having at least some college education (BA, MA, PhD or equivalent), which is slightly higher when compared to the UK national average [47]. Indeed, several studies have observed that online samples report higher education than one finds in representative samples. Nevertheless, our study’s educational composition is similar to a recent online research on COVID-19 knowledge in the United States and the United Kingdom [48] and 1 study conducted on COVID-19 prevention [42]. In this study, we did explore the effect of education on participants’ view time. We first assumed that participants with higher education are more receptive to health education campaigns and more likely to seek health information [49,50]. Our results reveal there was no statistically significant difference in terms of engagement time across the different educational levels. Thus, although the high education status may be a limitation, we do not believe this has significantly affected our results and conclusions.

### Conclusion

SAS videos demonstrate potential for engaging diverse audiences and thereby enhancing the distribution of health education messages. Designed to be emotionally arousing and culturally neutral, SAS videos can facilitate public health efforts to promote healthy behaviors and meet audiences where they are across the media landscape. The evidence from this study demonstrates promising engagement with the SAS health messaging modality, across diverse audiences. As these audiences spend increasingly more time online, the need for innovative approaches to engaging them also increases. Even the most accurate and clear health messages have little value if they fail to reach their target viewers. For this reason, researchers and health communicators of the future will need to understand how to optimally engage their audiences and research in this field should be a high priority.

## Appendix

Multimedia Appendix 1 Supplementary Materials.

Multimedia Appendix 2 CONSORT-EHEALTH checklist (V. 1.6.1).

## Abbreviations

RCT: randomized controlled trial
SAS: short, animated story-based
