# Supplementary material for: Participant Engagement and Reactance to a Short, Animated Video About Added Sugars: Web-based Randomized Controlled Trial
Source: JMIR Public Health Surveill. 2022 Jan 24;8(1):e29669. doi: 10.2196/29669 (PMC8822418; doi:10.2196/29669)
Supplement: Multimedia Appendix 1 [file publichealth_v8i1e29669_app1.docx]

**Supplement**

**Sample size**

We calculated the sample size needed for pairwise comparisons between three groups using a oneway analysis of variance (ANOVA). The formula to calculate the sample size is [1]:

| 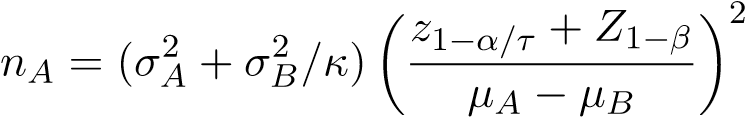 | (1) |
| --- | --- |

where κ = 1, which is the matching ratio, µA and µB are the group A and B means, σA and σB are the group A and B standard deviations, α = 0.05 is the Type-I error, β = 0.20 is the Type-II error, z is the quantile function, and τ = 2 is the number of comparisons to be made. We assumed a mean of µA = 2.0 and µB = 2.1 for the control and treatment groups, respectively. (In other words, we expect, on average, that the control group will agree with 2 out of the 5 items and the treatment group with 2.1 of the 6 items.) We selected σA = 0.85 and σ = 0.95. This calculation gives a sample size of n = 1276. For a 5-way comparison, the sample size is n = 1630. To ensure we have sufficient power, we will select a sample size of n = 4000.

**Table S1 *- Trait reactance items based on the Hong Psychological Reactance Scale [2]***

| ***Item*** | ***Factor*** |
| --- | --- |
| 6. I become frustrated when I am unable to make free and independent decisions.  7. It irritates me when someone points out things which are  obvious to me.  8. I become angry when my freedom of choice is restricted. | Emotional response to  restricted choice |
| 1. Regulations trigger a sense of resistance in me.  2. I find contradicting others stimulating.  3. When something is prohibited, I usually think, "That’s exactly what I am going to do." | Reactance to compliance |
| 11. I resist the attempts of others to influence me.  12. It makes me angry when another person is held up as a role model for me to follow.  13. When someone forces me to do something, I feel like doing the opposite. | Resisting influence from  others |
| 5. I consider advice from others to be an intrusion.  9. Advice and recommendations usually induce me to do just the opposite. | Reactance to advice and  recommendations |

**Table S2 – *Heterogeneous Effects: Trait Reactance Proneness***

| **Engagement Time** | **Coefficient** | **Robust Std.Err.** | **P-value** | **95% Confidence Interval** | |
| --- | --- | --- | --- | --- | --- |
| ***Trait Proneness Reactance*** | -11.991 | 10.709 | .263 | -33.004 | 9.021 |
| ***Narrator*** |  |  |  |  |  |
| Mother | 15.417 | 43.512 | .723 | -69.964 | 100.799 |
| Doctor | -2.236 | 44.709 | .960 | -89.967 | 85.495 |
| ***Narrator*Trait Proneness Reactance*** |  |  |  |  |  |
| Mother | -3.726 | 14.436 | .796 | -32.054 | 24.600 |
| Doctor | 0.437 | 14.870 | .977 | -28.742 | 29.617 |
| ***Education*** |  |  |  |  |  |
| High School | 20.618 | 21.971 | .348 | -22.496 | 63.732 |
| BA, some College | 16.881 | 20.625 | .413 | -23.590 | 57.354 |
| MA/ PhD | 25.871 | 21.369 | .226 | -16.060 | 67.802 |

**Notes** – The study sample consists of 1576 participants, of which 790 belong to the Content Placebo group and 786 to the Placebo group. For this analysis, we considered only those participants who chose to watch the video (n = 1047). The dependent variable is “Engagement Time” which is the view time of the SAS video expressed in seconds (min=0, max=222). Narrator and Education Status are categorical questions. Trait Proneness is participants’ trait reactance proneness mean score and is a continuous variable (min=0, max=5).

**References**

1. Rosner B. Fundamentals of biostatistics. 7th ed. Boston, MA: Cengage Publishers; 2010.

2. Hong S-M, Faedda S. Refinement of the Hong psychological reactance scale. Educational and Psychological Measurement. 1996;56:173–82.
